# Activation of Piezo1 or TRPV2 channels inhibits human ureteral contractions via NO release from the mucosa

**DOI:** 10.3389/fphar.2024.1410565

**Published:** 2024-06-26

**Authors:** Jianing Liu, Cong Wang, Wenyu Wang, Ning Ding, Jiaxin Liu, Hanwen Liu, Jiliang Wen, Wendong Sun, Shulu Zu, Xiulin Zhang, Jieke Yan

**Affiliations:** ^1^ Department of Kidney Transplantation, Multidisciplinary Innovation Center for Nephrology, The Second Hospital of Shandong University, Jinan, Shandong, China; ^2^ Department of Urology, The Second Hospital of Shandong University, Jinan, Shandong, China; ^3^ Department of Radiology, Shandong Provincial Hospital Affiliated to Shandong First Medical University, Jinan, Shandong, China

**Keywords:** mechano-sensitive channels, mucosa, Piezo1 channel, TRPV2 channel, ureteral motility

## Abstract

We aimed to investigate the expression and motor modulatory roles of several mechano-sensitive channels (MSCs) in human ureter. Human proximal ureters were obtained from eighty patients subjected to nephrectomy. Expression of MSCs at mRNA, protein and functional levels were examined. Contractions of longitudinal ureter strips were recorded in organ bath. A fluorescent probe Diaminofluoresceins was used to measure nitric oxide (NO). RT-PCR analyses revealed predominant expression of Piezo1 and TRPV2 mRNA in intact ureter and mucosa. Immunofluorescence assays indicate proteins of MSCs (Piezo1/Piezo2, TRPV2 and TRPV4) were mainly distributed in the urothelium. Ca2+ imaging confirmed functional expression of TRPV2, TRPV4 and Piezo1 in cultured urothelial cells. Specific agonists of Piezo1 (Yoda1, 3–300 μM) and TRPV2 (cannabidiol, 3–300 μM) attenuated the frequency of ureteral contractions in a dose-dependent manner while the TRPV4 agonist GSK1016790A (100 nM–1 μM) exerted no effect. The inhibitory effects of Piezo1 and TRPV2 agonists were significantly blocked by the selective antagonists (Dooku 1 for Piezo1, Tranilast for TRPV2), removal of the mucosa, and pretreatment with NO synthase inhibitor L-NAME (10 μM). Yoda1 (30 μM) and cannabidiol (50 μM) increased production of NO in cultured urothelial cells. Our results suggest that activation of Piezo1 or TRPV2 evokes NO production and release from mucosa that may mediate mechanical stimulus-induced reduction of ureter contractions. Our findings support the idea that targeting Piezo1 and TRPV2 channels may be a promising pharmacological strategy for ureter stone passage or colic pain relief.

## Highlights

• Mechano-sensitive channels (Piezo1/2, TRPV2 and TRPV4) are expressed in human ureters.
• Activation of Piezo1 or TRPV2 attenuated the frequency of ureteral contractions.
• NO release from the mucosa mediates Piezo1 or TRPV2 activation induced inhibition.

## 1 Introduction

The function of the ureter is to ensure unidirectional transport of urine from the kidney to bladder. This activity is accomplished by ureteral peristalsis, which is regulated by various myogenic and neurogenic mechanisms (Canda et al., 2007; Osman et al., 2009). Clarification of the mechanisms involved in regulation of ureteral contraction and relaxation is critical for the development of pharmacological agents that can effectively facilitate urinary stone passage, alleviate stent-associated symptoms, and relieve colic pain.

The neurogenic regulation mechanisms associated with ureteral contractions mainly involve sympathetic, para-sympathetic, and sensory nerves (Morita et al., 1987; Patacchini et al., 1998). In addition, non-adrenergic non-cholinergic (NANC) components from neuronal and non-neuronal sources play important regulatory roles (Santicioli and Maggi, 1998). Adenosine triphosphate (ATP) has been identified as an important NANC agent that triggers ureteral contractions. Furthermore, prostaglandins are reported to induce contraction (PGF2) or relaxation (PGE1, PGE2) of the ureter in animals and humans (Morita et al., 1994). Nitric oxide (NO) is a major inhibitory neurotransmitter causing ureteral relaxation (Iselin et al., 1997).

The urothelium is an important source of NANC components and plays a crucial regulatory role in detrusor contractions (Birder and Andersson, 2013; Sellers et al., 2018). Urothelial cells express various receptors and ion channels that act to sense changes in the extracellular environment and respond to chemical, mechanical and thermal stimuli by releasing various factors, such as ATP, NO, and acetylcholine (Ach) (Canda et al., 2007; Birder and Andersson, 2013; Shabir et al., 2013). Mechanical stimuli serve as important modulators of ureteral contractions, since the ureter faces changes in urine flow, stretch, distension and pressure under specific conditions, such as obstructions, stones or ureteral stents. Stent insertion initially induces an increase in peristaltic activity, which is subsequently reduced or terminated (Venkatesh et al., 2005). Moreover, after removal of an obstruction, a brief or long-lasting period of diminished or complete lack of contraction is often observed (Vereecken, 1976). However, the mechanisms underlying mechanical stimuli-mediated regulation of ureter contractions remain elusive.

Mechano-sensitive channels (MSCs) on urothelial cells are responsible for the detection and transduction of mechanical stimuli. Several classes of MSCs have been implicated in urothelial mechano-transduction. These channels include transient receptor potential vanilloid (TRPV2, TRPV4) (Wu et al., 2021), epithelial Na^+^ (Wang et al., 2003), and Piezo1/Piezo2 (Miyamoto et al., 2014; Marshall et al., 2020; Dalghi et al., 2021). Among the MSCs identified in the urothelium, only Piezo1/Piezo2 channels have been classified as *bona fide* mechano-sensors. Piezo1 and Piezo2 are nonselective cation channels activated by various mechanical stimuli, including stretch, hydrostatic pressure, and laminar flow (Coste et al., 2012). Piezo1 is additionally activated by the chemical agonist Yoda1 (Botello-Smith et al., 2019). However, the lack of a selective agonist for Piezo2 has limited its functional characterization to date. While the functions of TRPV2 and TRPV4 in bladder urothelial mechano-transduction have been extensively studied, their potential regulatory roles in ureteral contractions are yet to be elucidated (Everaerts et al., 2010; Dalghi et al., 2021; Gailly and Devuyst, 2021).

The main objective of this study was to investigate the expression patterns of MSCs at mRNA, protein and functional levels in human ureter. The modulatory roles of Piezo1, TRPV2 and TRPV4 in ureter mobility were further explored using the agonists and antagonists in isolated human proximal ureter strips in organ bath.

## 2 Materials and methods

### 2.1 Ureter strip preparation

All experimental procedures were approved by the Ethics Committee of the Second Hospital, Cheeloo College of Medicine, Shandong University (Jinan, Shandong, China) (KYLL-2020 (LW)-084). Human ureter tissues were obtained from eighty patients (42 female, 38 male; mean age, 52.5 ± 10.7 years; range, 36–61 years) subjected to nephrectomy. All patients provided written informed consent before participating in the study. Ureteral tissues were isolated from radical nephrectomy specimens. All tissue specimens appeared macroscopically normal, with no signs of tumor, obstruction, inflammation or other changes. The proximal ureter, approximately 6 cm in length, was excised 2 cm away from the pelvis. Following excision, ureteral tissues were immediately transported to the laboratory, where the surrounding vascular, adipose and connective tissues were carefully removed. For urothelium-intact strips, a segment of ureter was first opened and then was longitudinally cut into two parts. For denuded strip preparation, one segment of ureter was opened with the urothelium side up and the mucosa carefully removed with fine forceps under a microscope. Longitudinal segments (length, 10 mm) of intact or denuded ureters were isolated from each ureter sample for organ bath experiments. Small sections of the tissues were employed for polymerase chain reaction (PCR) analyses.

### 2.2 Organ bath experiments

Organ bath experiments were conducted as described previously by our group (Liu et al., 2021). Briefly, whole-layer or mucosa-free ureteral specimens were placed in warm Krebs solution composed of NaCl 118 mM, KCl 4.7 mM, CaCl_2_ 1.9 mM, MgSO_4_ 1.2 mM, NaHCO_3_ 24.9 mM, KH_2_PO_4_ 1.2 mM, and glucose 11.7 mM, pH 7.4. Longitudinal ureteral strips (10 ± 1.5 mm in length, 2–3 mm in width) were tied at each end using a fine thread and mounted in a vertical organ bath in Krebs solution (10 mL). Subsequently, Krebs solution was heated to 37°C in a circulating warm water bath and continuously gassed with 95% O_2_ and 5% CO_2_. The longitudinal tension of the strips was continuously recorded with an isometric transducer and processed using LabChart 7 software (AD Instruments Pty Ltd., New South Wales, Australia). The preparations were stretched to 0.5 g and allowed to equilibrate for 20–30 min until the appearance of spontaneous contractions. In slices with no spontaneous contractions, NKA (10–30 nM) was applied to initiate contractions. All the strips showed a spontaneous or NKA-evoked response, indicative of good viability.

### 2.3 Reverse transcription-quantitative polymerase chain reaction

Ureter specimens were frozen in liquid nitrogen and stored at −80°C prior to experimental use. Total RNA was extracted using the RNA Simple Total RNA kit (Tiangen, Beijing, China) and RNA concentrations quantified with an ultraviolet spectrophotometer (DeNovix, United States). Reverse transcription was conducted using a SPARKscript II RT plus Mix kit (Sparkjade, Qingdao, China) according to the manufacturer instructions, and complementary DNA amplified (40 cycles of denaturation for 15 s at 95°C and primer annealing and elongation for 30 s at 60°C). Quantitative reverse transcription polymerase chain reaction (RT-qPCR) was carried out using a SYBR™ Green qPCR Mix (Sparkjade) and QuantStudio™ 5 system (Thermo Fisher, Waltham, MA, United States). Specific primers for β-actin, TRP and Piezo channel genes were synthesized by BioSune (Shanghai, China). The primer sequences are listed in Table 1. Relative expression was measured using the 2^−ΔΔCT^ method.

**TABLE 1:** Oligonucleotide primer sets for quantitative real-time PCR (RT-PCR).

Name	Sequence (5′–3′)	Length	Tm
TRPV2 F	TCG​CTG​TAT​GAC​CTG​GCT​TC	20	62
TRPV2 R	GCT​CCA​AAA​CGA​CCA​TTC​GG	20	62
TRPV4 F	TCT​CAC​CGC​CTA​CTA​CCA​GC	20	64
TRPV4 R	GTA​GAG​GGC​TGC​TGA​GAC​GA	20	62
Piezo1 F	ACT​TTC​CCA​TCA​GCA​CTC​GG	20	64
Piezo1 R	CCA​CGA​AGT​CCT​TGA​GAC​CC	20	64
Piezo2 F	ACT​GCT​GGG​AAA​GTC​GTT​GT	20	60
Piezo2 R	TTG​GGT​GGA​ACT​GCC​TCT​TG	20	60
β-Actin F	CAT​GTA​CGT​TGC​TAT​CCA​GGC	21	57.6
β-Actin R	CTC​CTT​AAT​GTC​ACG​CAC​GAT	21	55.6

F, forward; R, reverse; T_m_, melting temperature.

### 2.4 Immunofluorescence staining

Sections of ureter tissue (5 μm) were fixed in 4% paraformaldehyde for 15 min following three washes with PBS. Next, sections were blocked with 5% normal goat serum for 30 min and incubated with two mixed primary antibodies (specified in Table 2) at 4°C overnight on a shaker. After washing with phosphate-buffered saline (PBS), sections were incubated with the appropriate secondary antibodies for 1 h at room temperature, specifically, ABflo^®^ 488-conjugated goat anti-mouse IgG (H + L, diluted 1:100 in PBS) and ABflo^®^ 594-conjugated goat anti-rabbit IgG (H + L, diluted 1:100 inPBS). Fluorescent images were captured using an Olympus BX53 inverted fluorescence microscope.

**TABLE 2:** Primary antibodies used in immunohistochemistry experiments.

Antibody	Host	Supplier	Code	Dilution
TRPV2	Rabbit	Sigma-Aldrich (Munich, Germany)	SAB1101376	1:100
TRPV4	Rabbit	Novus Biologicals (Littleton, CO, United States)	NBP2-41262	1:100
Piezo1	Rabbit	Affinity Biosciences LTD. (JiangSu, China)	DF12083	1:100
Piezo2	Rabbit	Alomone Labs (Jerusalem, Israle)	APC-090	1:100
AE1/AE3	Mouse	Abcam (Cambridge, United Kingdom)	Ab80826	1:200

### 2.5 Urothelial cell cultures

Urothelial cell cultures were established as described previously (Wen et al., 2021). The ureter was opened and incubated in Dispase (2.5 mg/mL, Worthington Biochemical Co., Lakewood, NJ, United States) overnight at 4°C with the urothelium side up. Urothelial cells were gently scraped, placed in trypsin (0.25% wt/vol, Sigma) for 10–15 min at 37°C, and dissociated via trituration. Cells were suspended in MEM containing 10% FBS and centrifuged at 416 g for 5 min. Subsequently, cells were suspended in urothelial cell medium (UCM; ScienCell, San Diego, CA, United States) with 1% PSF, re-centrifuged, and resuspended in fresh medium. At 48–96 h after dissociation, cells were plated on poly-L-lysine-coated glass coverslips and used for Ca^2+^ imaging.

### 2.6 Ca^2+^ imaging

Cultured urothelial cells on glass coverslips were loaded with 2 μM Fura-2-acetoxymethyl ester (Fura 2-AM; Dojindo Laboratories, Tongren, Japan) for 30 min. Fura 2-AM was dissolved in Hank’s balanced salt solution containing (in mM): 138 NaCl, 5 KCl, 0.3 KH_2_PO_4_, 4 NaHCO_3_, 2 CaCl_2_, 1 MgCl_2_, 10 HEPES, and 5.6 glucose, pH 7.4. Ca^2+^ imaging was performed as described previously by our group (Wen et al., 2018). Briefly, coverslips were placed in a recording chamber. Fura 2-AM was alternately excited with ultraviolet light at 340 nm and 380 nm and the fluorescence emission was detected at 510 nm using a computer-controlled monochromator. Wavelength selection, timing of excitation, and image acquisition were controlled using MetaFluor^®^ software (Molecular Devices, Sunnyvale, CA, United States). The ratio of the fluorescence signal measured at 340 nm to that measured at 380 nm was used to estimate the increase in [Ca^2+^]_i_. Ratio changes >0.1 indicated a significant increase in [Ca^2+^]_i_.

### 2.7 Single cell mechanical stimulation

Mechanical stimulation of single urothelial cell was performed as described by our previous study (Zhao et al., 2021). The glass micropipettes with a closed and rounded tip (∼2 μm) were used to deflect the plasma membrane. The movement of glass micropipettes was controlled by a motorized MP-285 micromanipulator (Sutter Instruments, Novato, CA, United States). When the tip is close to the cell, the micropipette was dropped in 2 μm increments to induce the membrane deflection.

### 2.8 Detection of NO production with fluorescent indicator DAF-2 DA

NO production in urothelial cells was detected by fluorescence NO indicator, 4,5-diaminofluorescein diacetate (DAF-2 DA) as described previously (Pajolla et al., 2009). Briefly, the cells on coverslips were incubated with 10 μM DAF-2 DA (in 1% DMSO) for 30 min in darkness. Then, cells were rinsed twice with PBS. Direct visualization of the NO production with this fluorescent indicator was performed in combination with Inverted fluorescence microscope (Olympus IX73, Japan). Semi-quantitative analysis of NO-induced fluorescence (NO_IF_) was performed using imaging analysis software (ImageJ, v.1.54, NIH, United States) and measured 10 min after the agonists application and expressed as fluorescence arbitrary units. The mean value of the three coverslips for each group was quantified and analyzed.

### 2.9 Chemicals

The chemicals used in this study were as follows: Acetylcholine iodide (Ach), Adenosine triphosphate (ATP, Sigma-Aldrich, Inc., St. Louis, MO, United States), Cannabidiol (CBD; a TRPV2 agonist, Tocris Cookson, Bristol, United Kingdom), Dooku1 (a Yoda1 antagonist), GSK1016790A (GSK, a selective TRPV4 agonist; Sigma-Aldrich, Inc.), HC-067047 (a selective TRPV4 antagonist), Indomethacin (a COX1/2 inhibitor), L-NAME hydrochloride (a NOS inhibitor), Neurokinin A, Prostaglandin El (PGE1), Prostaglandin E2 (PGE2), S-Nitroso-N-acetyl-DL-penicillamine (SNAP, a nitric oxide donor), Tranilast (Tra; a specific TRPV2 antagonist), and Yoda1 (a selective Piezo1 agonist). Unless otherwise stated, chemicals were acquired from MCE company (MCE, New Jersey, United States). The stock solution of NKA, ATP, L-NAME hydrochloride was prepared in distilled water and those of other chemicals prepared in dimethyl sulfoxide (DMSO). DMSO (final concentration of 0.1%) had no significant effects on the activity of ureteral strips. Near-saturation concentrations were selected for each agonist and antagonist based on published reports.

### 2.10 Statistical analysis

All data are presented as means ± S.E.M and analyzed using Prism 8.0.2 (GraphPad, San Diego, CA, United States). The effects of various drugs were evaluated as % change in contraction frequency from baseline. The frequency was measured at 5 min intervals immediately before and after drug application. Concentration–response curves were fitted with the Hill equation as follows: % inhibition = [MAX_inhibition_/(drug concentration + IC_50_)] n, whereby MAX_inhibition_ = maximal% inhibition, drug concentration = concentration of the agonist, IC_50_ = half‐maximal concentration, and n = the Hill coefficient. Statistical significance was evaluated with one‐sample t‐test or paired two‐tailed *t*‐test with layered Bonferroni *post hoc* test for multiple comparisons when appropriate. Differences were considered statistically significant at *p* < 0.05.

## 3 Results

### 3.1 Expression of mechano-sensitive channels in human ureter

**FIGURE 1:**
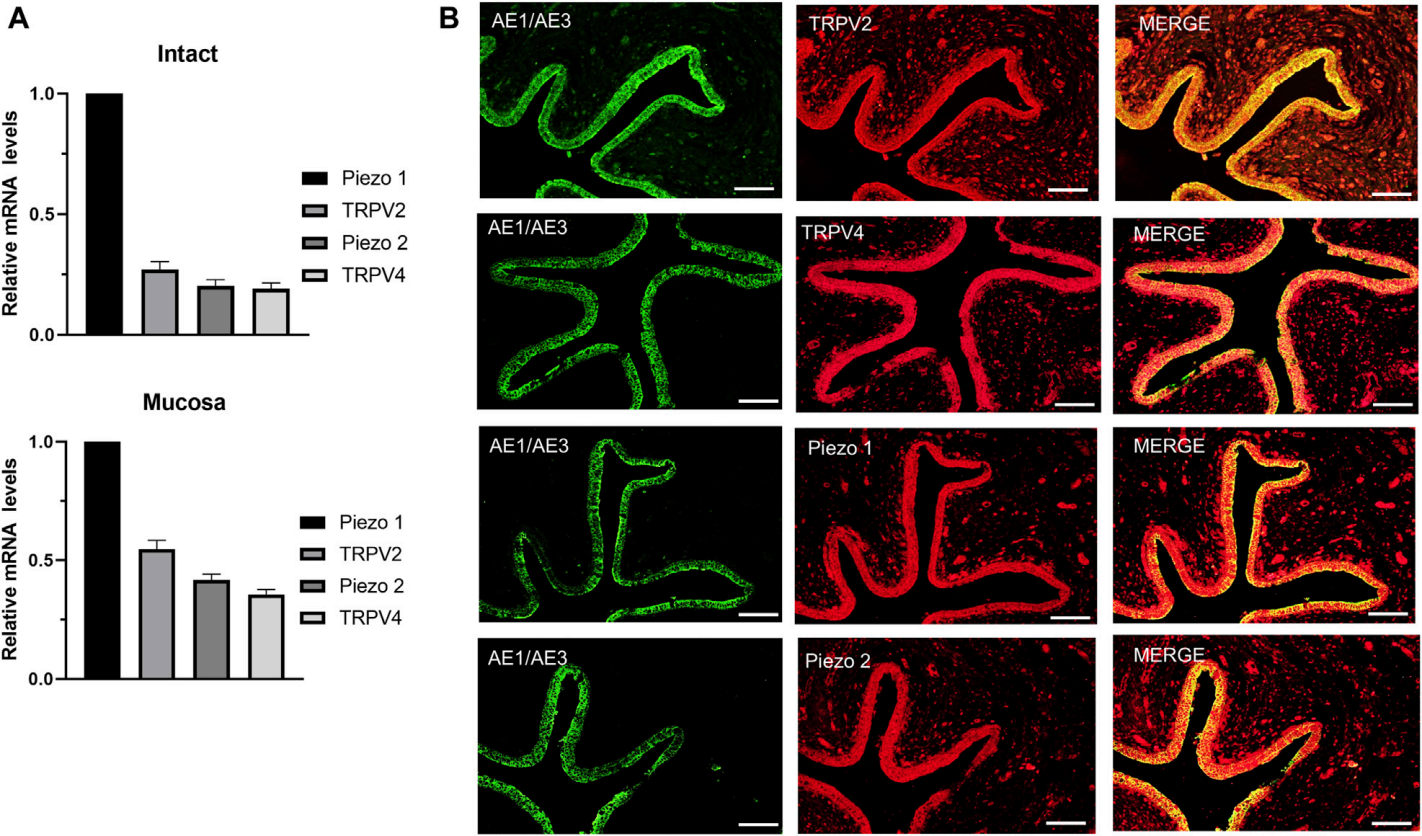
mRNA and protein expression patterns of mechano-sensitive channels in human ureter. **(A)** Relative mRNA expression of mechano-sensitive channels in intact human proximal ureter or mucosa. Total RNA was extracted from intact proximal ureter or mucosa for RT-qPCR. The primer sets for amplification of the four channels are shown in Table 1. In view of the significantly lower expression of the four channels relative to beta-actin, mRNA expression in each case was determined relative to Piezo1, which showed the highest expression. Summary data are the average values of six experiments. Expression patterns were similar in intact ureter and mucosa. **(B)** Immunofluorescence labeling showing that TRPV2, TRPV4, Piezo1, and Piezo 2 are mainly expressed on the urothelium of ureter. Urothelial cells were marked with the specific urothelial marker AE1/AE3. The scale bar = 100 μm.

TRPV2, TRPV4 and Piezo1/Piezo2 are known to function as mechano-sensors in various cell types. To ascertain whether these mechano-sensitive channels (MSCs) play a potential modulatory role in ureteral contractions, we initially investigated their expression patterns in human ureter samples. RT-qPCR experiments showed that the relative mRNA abundance was the highest for *Piezo1*, moderate for *TRPV2*, and lowest for *Piezo2* and *TRPV4*. The expression patterns were similar in tissues from the intact ureter and mucosa (Figure 1A).

Protein expression of the above MSCs in human ureter was examined via immunohistochemistry with specific antibodies (Table 2). Immunofluorescence of the four channels (Figure 1B middle panels) was mainly localized in urothelial cells stained with the marker AE1/AE3 (left panels). Less expression was observed in sub-urothelial layers.

**FIGURE 2:**
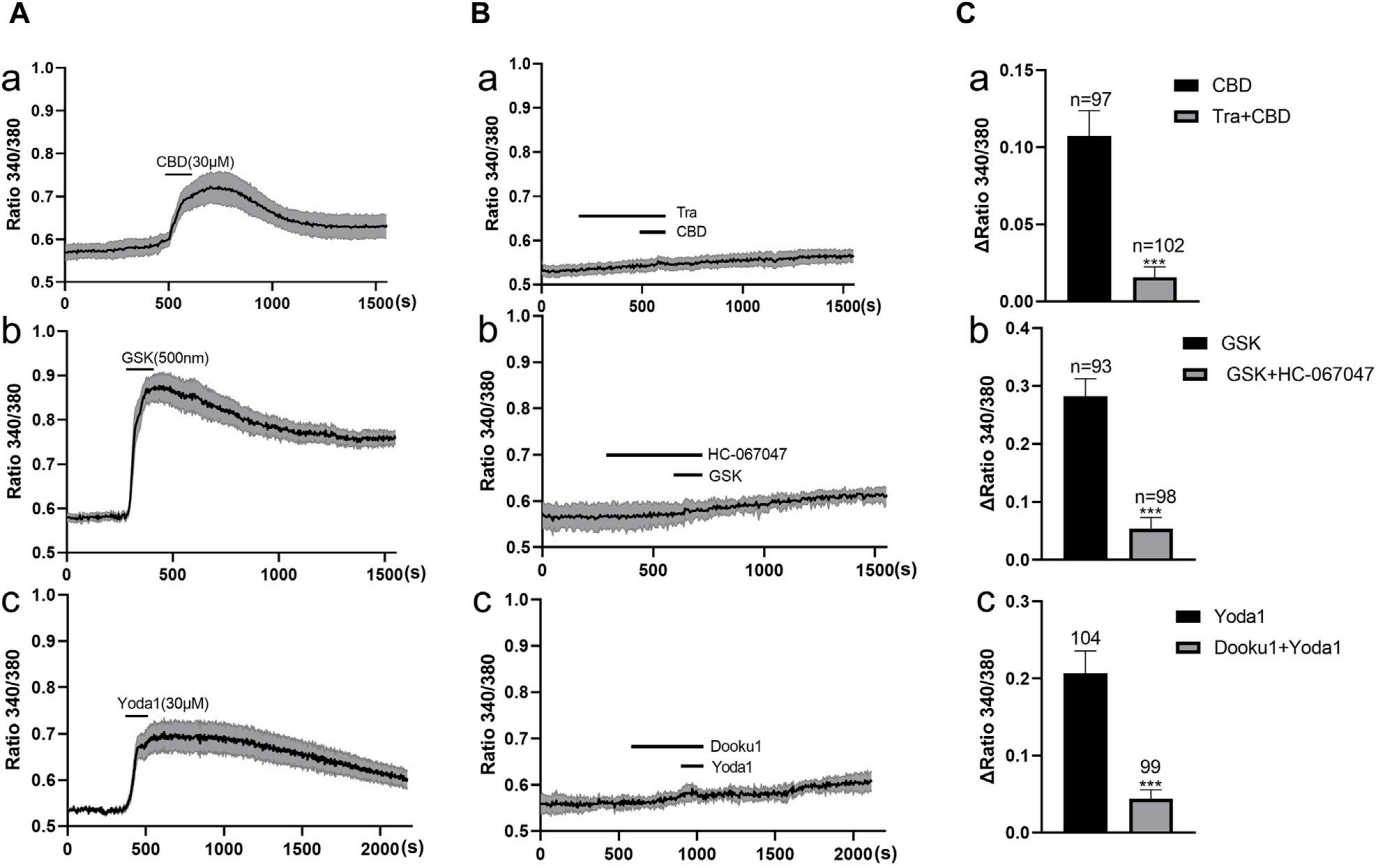
Functional expression of TRPV2, TRPV4 and Piezo1 channels in primary cultured human ureter urothelial cells. **(A)** The TRPV2 agonist cannabidiol (CBD 30 μM, a), TRPV4 agonist GSK (500 nM, b), and Piezo1 agonist Yoda 1 (30 μM, c) elicited a remarkable increase in [Ca^2+^]_i_. Each agonist was applied for 40–60 s. **(B)** Typical traces demonstrating blockage of the increase in [Ca^2+^]_i_ induced by agonists of TRPV2 (a), TRPV4 (b) and Piezo1 (c) upon pretreatment with the corresponding antagonists. Each trace in **(A,B)** was obtained by averaging several cell responses from the same coverslip. The antagonist was applied 5 min prior to the agonist. **(C)** Summary data on the blocking effects of antagonists. The number above each bar indicates the cell number. ***p < 0.001 versus agonist alone. CBD, cannabidiol; GSK, GSK1016790A; Tra, Tranilast.

Functional expression of these channels in primary cultured human urothelial cells was examined with the Ca^2+^ imaging method (Figure 2A). A agonist of TRPV2, cannabidiol (CBD, 30 μM) (De Petrocellis et al., 2011), elicited an increase in [Ca^2+^]_i_ in human urothelial cells (*n* = 97 cells from 14 coverslips; Figure 2Aa). This effect was blocked significantly by pretreatment with Tranilast (10 μM), a TRPV2-specific antagonist (Figures 2Ba, Ca). Since no specific agonists of TRPV2 are commercially available, the contribution of TRPV2 in CBD evoked [Ca^2+^]_i_ increase was verified by utilizing siRNA mediated knockdown of TRPV2 expression in cultured urothelial cells. We found that TRPV2-siRNA treatment significantly reduced CBD evoked increase in [Ca^2+^]_i_ (Supplementary Figure S1). Specific agonist of TRPV4, GSK (500 nM), evoked an increase in [Ca^2+^]_i_ in human urothelial cells (*n* = 93 cells from 13 coverslips; Figure 2Ab), which was blocked by pretreatment with the TRPV4 antagonist HC-067047 (1 μM; Figures 2Bb, Cb). Yoda 1 (30 μM), a specific agonist of Piezo1 channels (Syeda et al., 2015), evoked a significant increase in [Ca^2+^]_i_ in human urothelial cells (*n* = 104 cells from 13 coverslips; Figure 2Ac), which was markedly blocked by pretreatment with the Piezo1-specific antagonist DooKu1 (10 μM; Figures 2Bc, Cc). The doses of the three agonists used were previously determined as the saturating concentrations (De Petrocellis et al., 2011; Syeda et al., 2015). Since no commercial chemical agonists are available for Piezo2, its functional expression could not be investigated.

**FIGURE 3:**
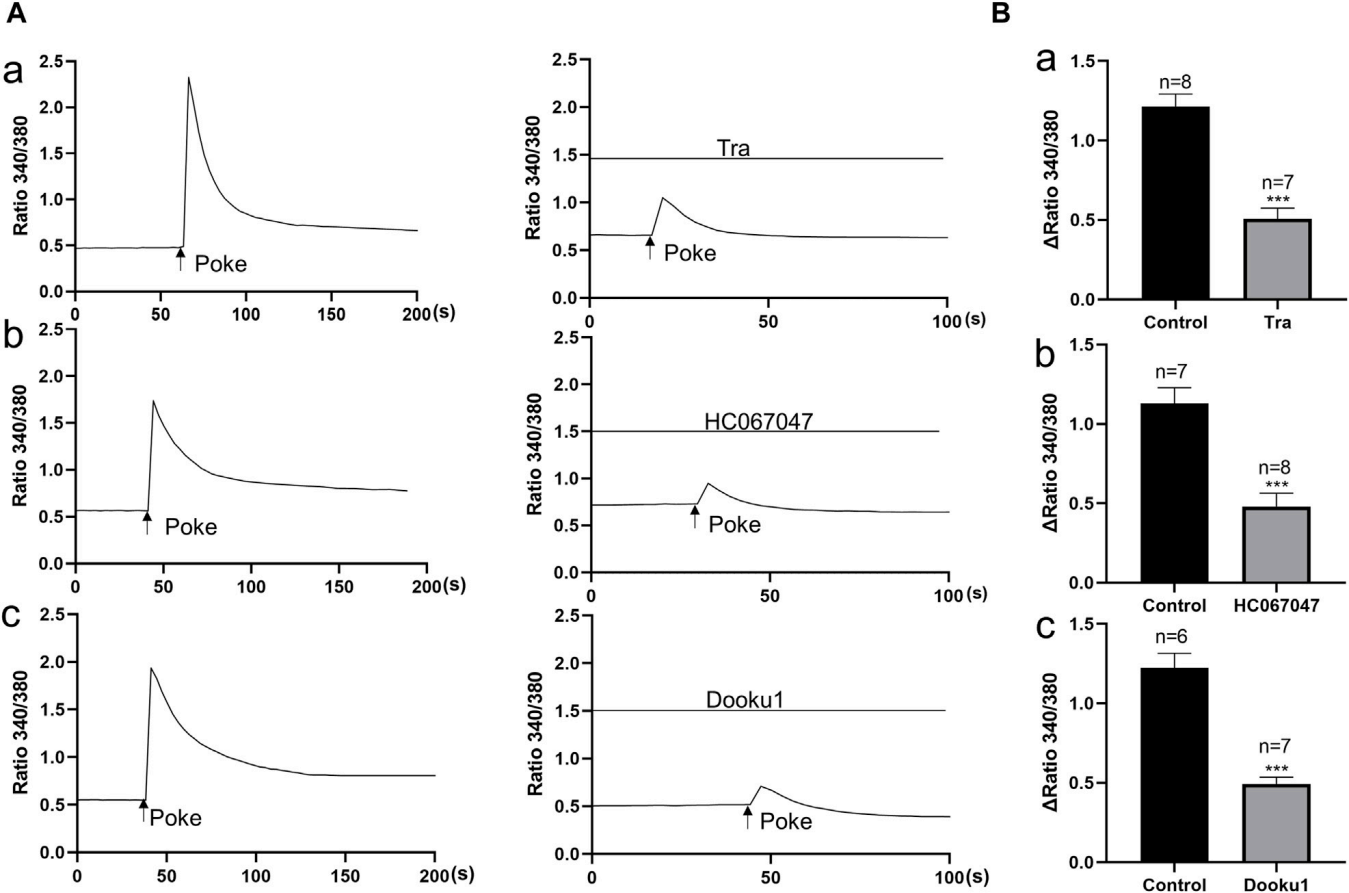
Antagonists of TRP V2, TRPV4 and Piezo1 channels inhibit mechanical stimulation induced increase in [Ca^2+^]_i_ in cultured urothelial cells. **(A)**, Typical traces demonstrating that pretreatment with the antagonist of TRPV2 (a, TRA, 10 μM), TRPV4 (b, HC067047, 1 μM) and Piezo1 (c, Dooku1, 10 μM) blocked poke induced increase in [Ca^2+^]_i_. To note, each typical trace in a–c is from one single cell recording. **(B)** Summary data for the blocking effects of the antagonists. n above each bar indicates the cell number examined. ***p < 0.001.

In order to examine the involvement of above functional active TRPV2, TRPV4 and Piezo 1 in mechanical responses of urothelial cells, the impacts of these channel antagonists on poke (applied via a glass micropipette) induced [Ca^2+^]_i_ increase was investigated. TRPV2 antagonist (Tranilast, 10 μM), TRPV4 antagonist (HC-067047, 1 μM) and Piezo1 antagonist (DooKu1, 10 μM) reduced the poke induced [Ca^2+^]_i_ increase by 58.7%, 57.6% and 59.7% respectively (Figure 3).

### 3.2 Modulatory role of mechano-sensitive channels in human proximal ureter contraction

**FIGURE 4:**
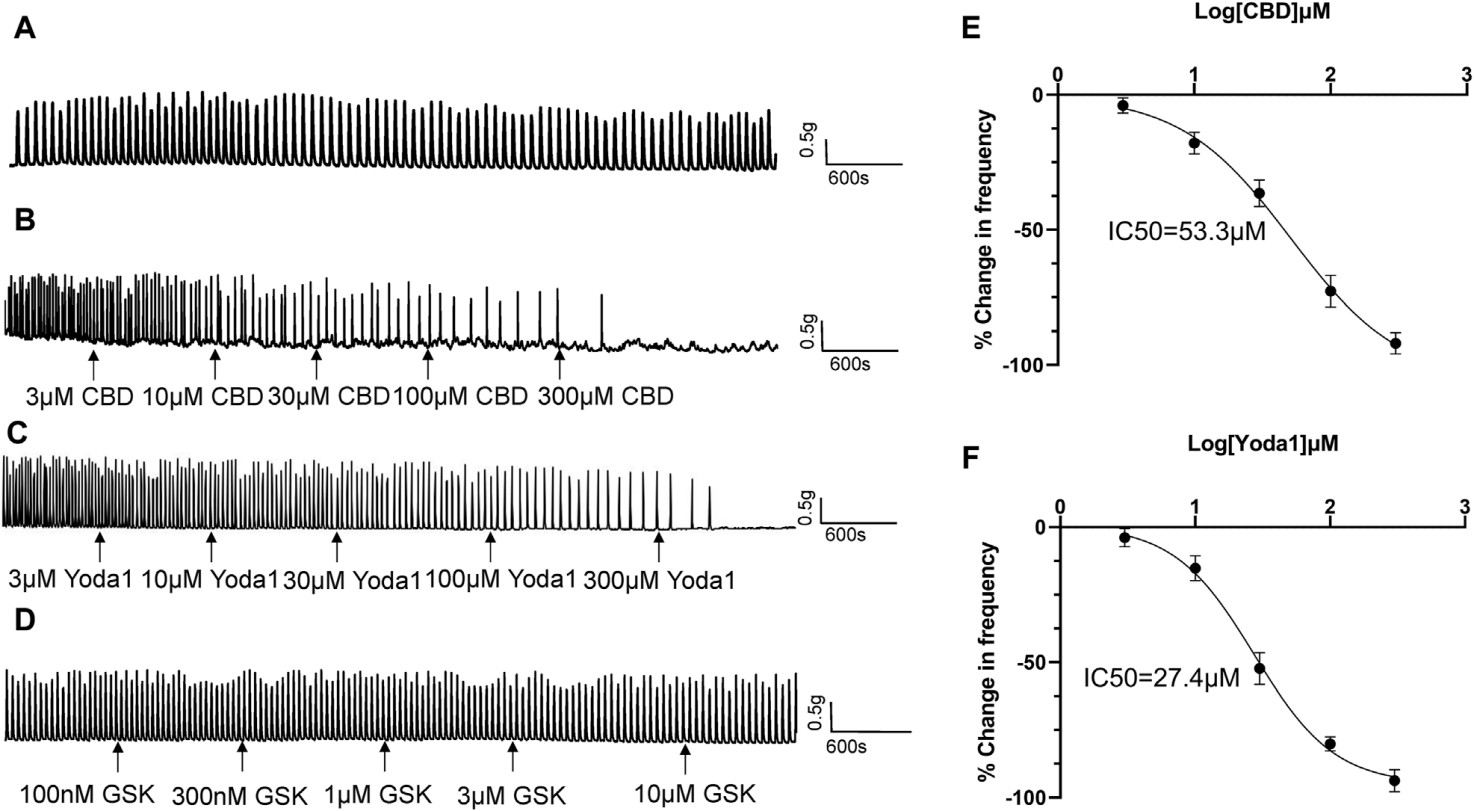
Inhibitory effects of TRPV2 and Piezo1 agonists on ureteral contractions. **(A)** A typical example showing vehicle control (0.01% DMSO) had no effect on the frequency of NKA-evoked contractions during 100 min recording period. **(B–D)** Representative traces showing that cumulative application of the TRPV2 agonist CBD (3–300 μM) **(B)**, Piezo1 agonist Yoda 1 (10–300 μM) **(C)** and TRPV4 agonist GSK(100 nM–10 μM) **(D)** induces a dose‐dependent reduction in the frequency of ureteral contractions. **(E)** Cumulative concentration-response curve (obtained from 14 strips of 6 patients) of CBD, yielding an IC_50_ value of 53.3 μM. **(F)** Cumulative concentration-response curve of Yoda1 (obtained from 14 strips from 6 patients), yielding an IC_50_ value of 27.4 μM. The drug effects are expressed as % change in the frequency (Hz) of ureteral contractions from baseline. CBD, cannabidiol.

Spontaneous contractions were observed in 25% (43 of 172) of the isolated proximal ureter strips. Since the frequency of ureteral contractions is an important component in physiological and pathological conditions, strips with no spontaneous contractions were treated with NKA (10–30 nM) to initiate contractions, with the aim of simultaneously observing the effects of the agonists of mechano-sensitive channels on both frequency and baseline tension. NKA-evoked and spontaneous phasic contractions had similar amplitude (1.7 ± 0.5 g, *n* = 79 vs. 1.6 ± 0.5 g, *n* = 46) and frequency (2.8 ± 0.3/min, *n* = 79 vs. 2.5 ± 0.3/min, *n* = 46). NKA-evoked contractions maintain consistent frequency over 100 min recording period (Figure 4A).

Cumulative addition of CBD to the bath (3–300 μM) decreased the frequency of ureteral contractions in a dose-dependent manner (Figures 4B, E). No significant reductions were observed in the magnitude and baseline tone of the phasic contractions. Analysis of the dose-response curve revealed an IC_50_ value of 53.3 μM for CBD (*n* = 14 strips of 6 patients; Figure 4E).

Cumulative addition of Yoda1 to the bath (3–300 μM) similarly decreased the frequency of ureteral contractions in a dose-dependent manner (Figures 4C, F). The magnitude and baseline tone of the contractions were not significantly reduced. Analysis of the dose-response curve revealed an IC_50_ value of 27.4 μM for Yoda1 (*n* = 14 strips of 6 patients; Figure 4F).

Although TRPV4 channels are functional in urothelial cells, unexpectedly, we observed no effect of the TRPV4 agonist GSK (100 nM–1 μM) on ureteral contractions (Figure 4D), even at the highest dose of 1 μM (about 50 times the EC_50_ value) (Thorneloe et al., 2008).

**FIGURE 5:**
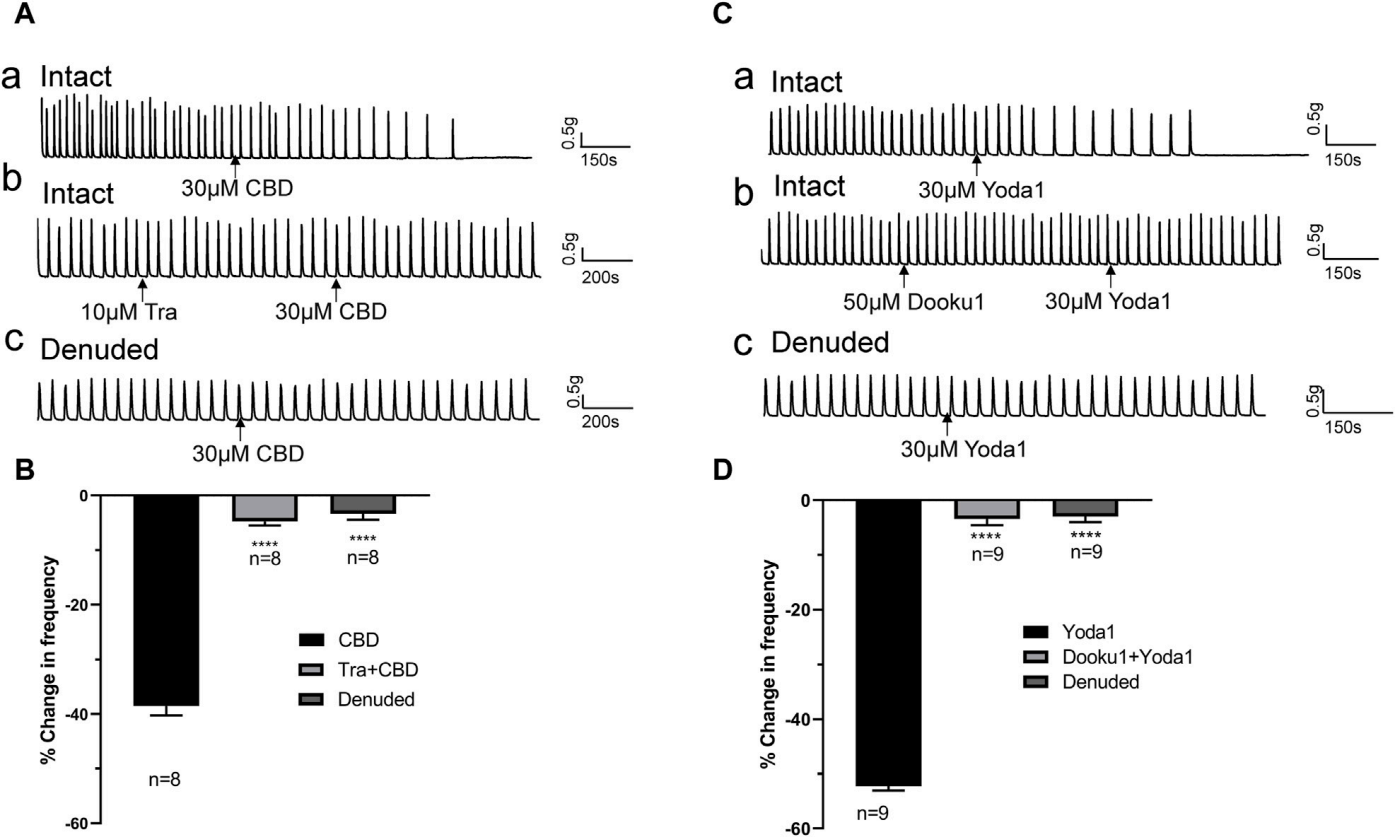
TRPV2 or Piezo1 channel agonist-induced inhibitory effects are blocked by the respective antagonists and removal of the mucosa. **(A,B)** Representative traces **(A)** showing significant attenuation of the inhibitory effects of TRPV2 agonist CBD (30 μM, a) upon pretreatment with the TRPV2 antagonist (Tra, 10 μM, b) or removal of the mucosa (c), and the summary data **(B)**. **(C,D)** Representative traces **(C)** showing significant blockage of the inhibitory effect of the Piezo1 agonist Yoda1 (30 μM, a) by the Piezo1 antagonist Dooku1 (50 μM, b) and removal of the mucosa (c), and the summary data **(D)**. The antagonist (Tra or Dooku1) was applied 5 min prior to treatment with the agonist (CBD or Yoda1). ****p < 0.0001 versus CBD or Yoda1 alone. n: the number of strips. CBD, cannabidiol; Tra, Tranilast.

The inhibitory effects of CBD (30 μM, *n* = 8 strips of 5 patients) were completely blocked by pretreatment with the TRPV2 antagonist Tranilast (Tra, 10 μM) 5–10 min prior to administration of CBD (Figures 5Ab, B). Similarly, the inhibitory actions of Yoda 1 (30 μM, *n* = 9 strips of 5 patients) were completely blocked by pretreatment with Dooku1 (50 μM) 5–10 min prior to administration of Yoda1 (Figures 5Cb, D).

### 3.3 Inhibitory effects of Piezo1/TRPV2 agonists are reduced in denuded ureter strips

The above experiments showed that Piezo1 and TRPV2 channels are mainly expressed in the mucosa (Figure 1B). To confirm the contribution of Piezo1 and TRPV2 channels in mucosa, CBD- and Yoda 1-induced effects were examined in mucosa‐devoid ureter strips. Our results showed that compared with the responses in intact ureters, CBD and Yoda 1-evoked inhibition was significantly attenuated in denuded strips (Figures 5Ac, Cc).

### 3.4 Release of NO from the mucosa mediates the inhibitory effects of TRPV2 and Piezo1 agonists

**FIGURE 6:**
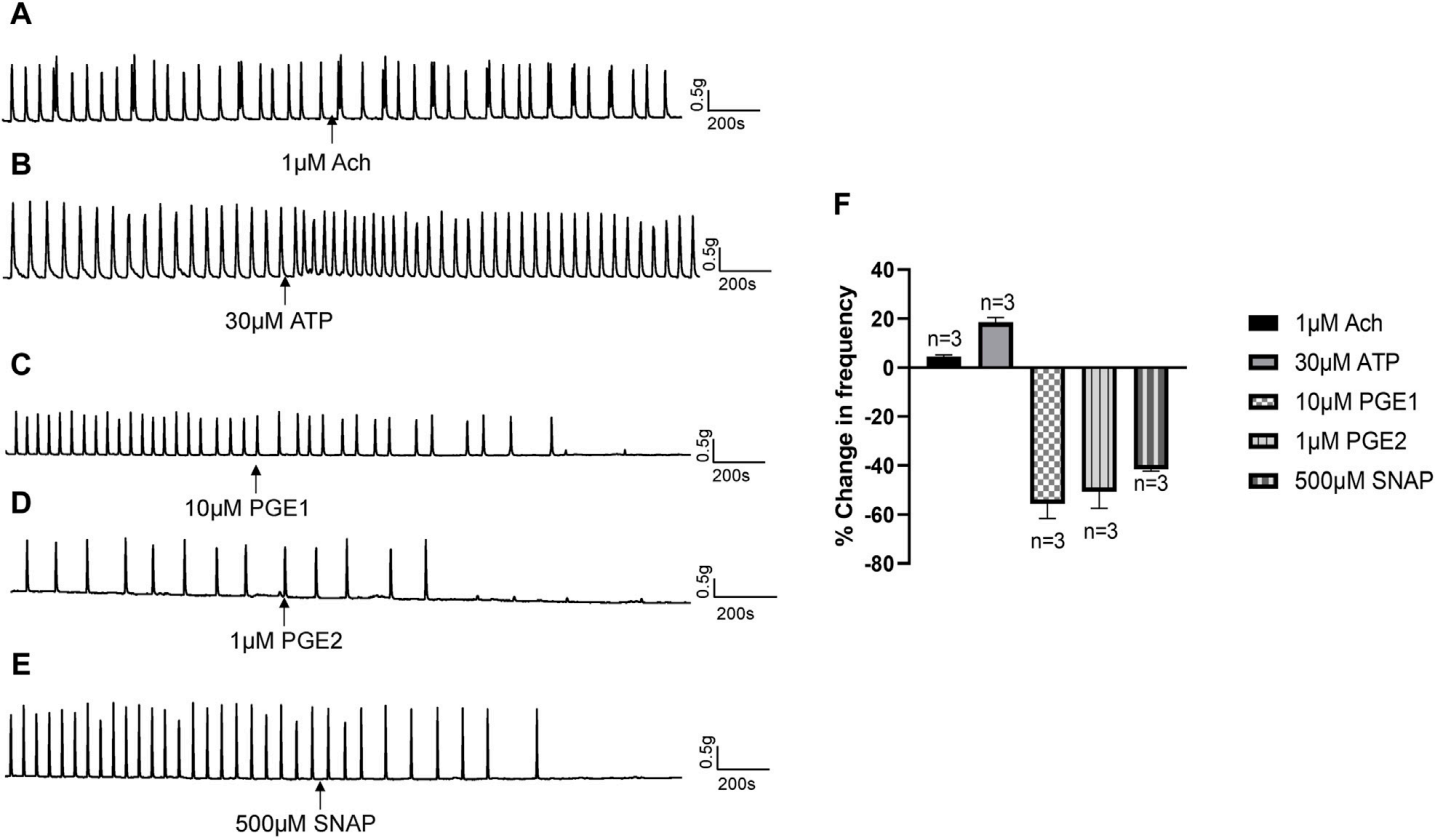
Effects of neurotransmitters released from urothelium on human ureteral contractions. **(A–E)** Representative traces showing that acetylcholine [Ach 1 μM; **(A)**] exerts no obvious effects while ATP [30 μM; **(B)**] produces an excitatory effect on ureteral contractions. PGE1 [10 μM; **(C)**], PGE2 [1 μM; **(D)**] and SNAP [500 μM; **(E)**] significantly reduced the frequency of ureteral contractions. **(F)** Summary data on the effects of neurotransmitters. Drug effects are expressed as % change in the frequency (Hz) from the baseline. n: the number of strips. Ach, acetylcholine; SNAP, S-Nitroso-N-acetyl-DL-penicillamine.

In response to chemical or mechanical stimulation, the urothelium releases neurotransmitters and modulators, such as ATP, acetylcholine (Ach), NO and prostaglandins, that exert excitatory or inhibitory effects on urinary tract motility (Guan et al., 2017). To identify the specific transmitters contributing to the inhibitory actions of Piezo1 and TRPV2 agonists, we initially examined the effects of Ach, ATP, PGE1, and PGE2 on isolated human ureters. Ach (1 μM) had no obvious effect on isolated ureteral contractions (Figure 6A), while ATP (30 μM) exerted a slight excitatory effect (Figure 6B). PGE1 (10 μM) along with PGE2 (1 μM) had obvious inhibitory effects (Figures 6C, D). Moreover, S-Nitroso-N-acetyl-DL-penicillamine (SNAP, 500 μM), a nitric oxide donor, exerted a significant inhibitory effect on ureteral contractions (Figure 6E). The summary data for all five agents are summarized in Figure 6F.

**FIGURE 7:**
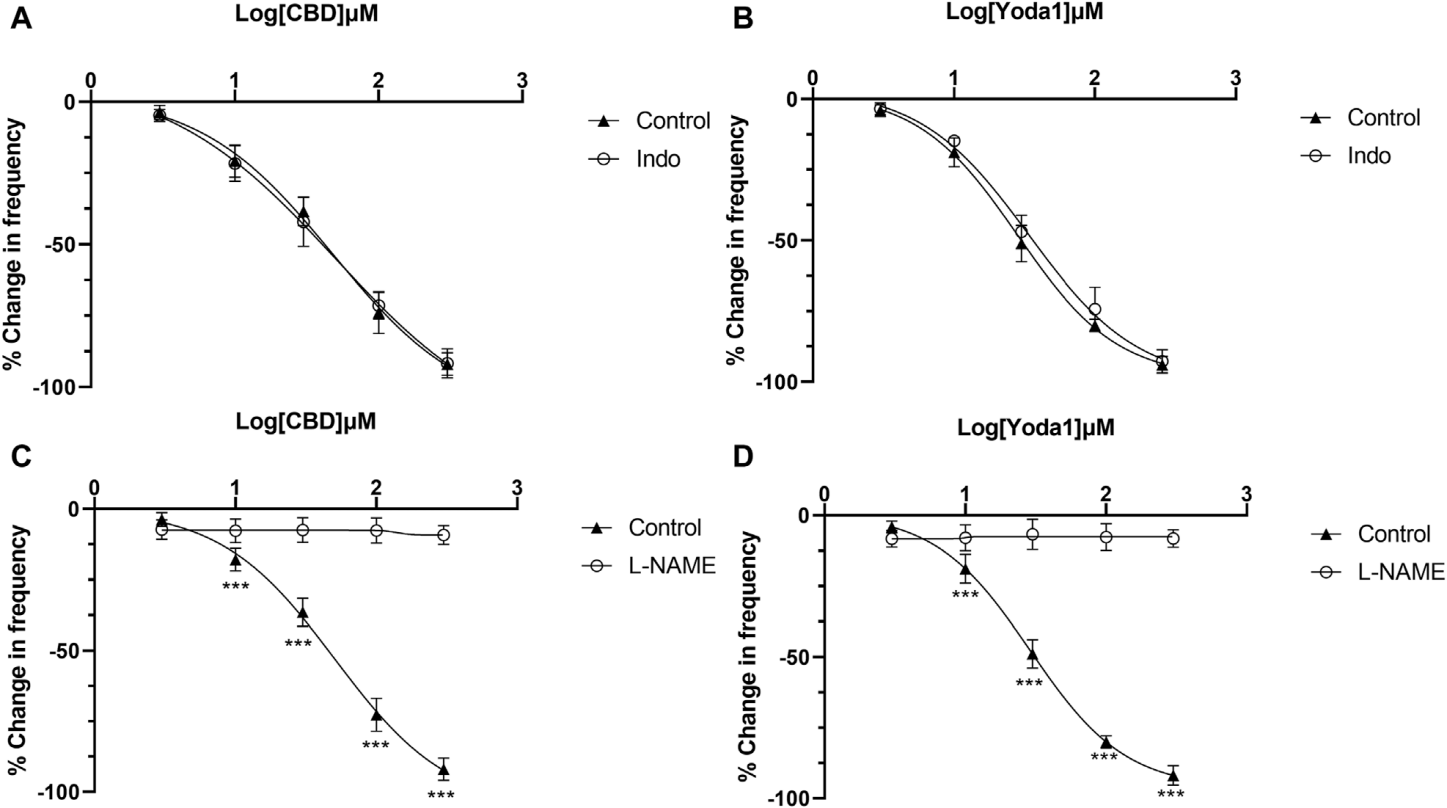
Release of NO mediates the inhibitory effects of TRPV2 and Piezo1 agonists. **(A,B)** Dose-response curves of CBD and Yoda1 showing that indomethacin does not influence the inhibitory effects of TRPV2 agonist **(A)** and Piezo1 agonist **(B)**. **(C,D)** Dose-response curves of CBD **(C)** and Yoda1 **(D)** showing that L-NAME, a NOS antagonist, significantly blocks the inhibitory effects of both TRPV2 and Piezo1 agonists (*t* tests). ***p < 0.001. Agonist effects are expressed as % change in the frequency (Hz) of ureteral contractions from the baseline. Each curve represents average data from 5–8 ureter strips. Indo: Indomethacin.

The above results indicate that NO or prostaglandins could serve as the potential neurotransmitters mediating the inhibitory effects of Piezo1 and TRPV2 agonists. In the presence of indomethacin (10 μM), no significant changes were observed in the dose-response curve of CBD (Figure 7A) or Yoda1 (Figure 7B), suggesting no blocking effects. In contrast, in the presence of L-NAME hydrochloride (50 μM), a non-selective nitric oxide synthase inhibitor, the dose-response curves of both CBD (Figure 7C) and Piezo1 (Figure 7D) were shifted to the right, indicative of a significant blocking effect.

**FIGURE 8:**
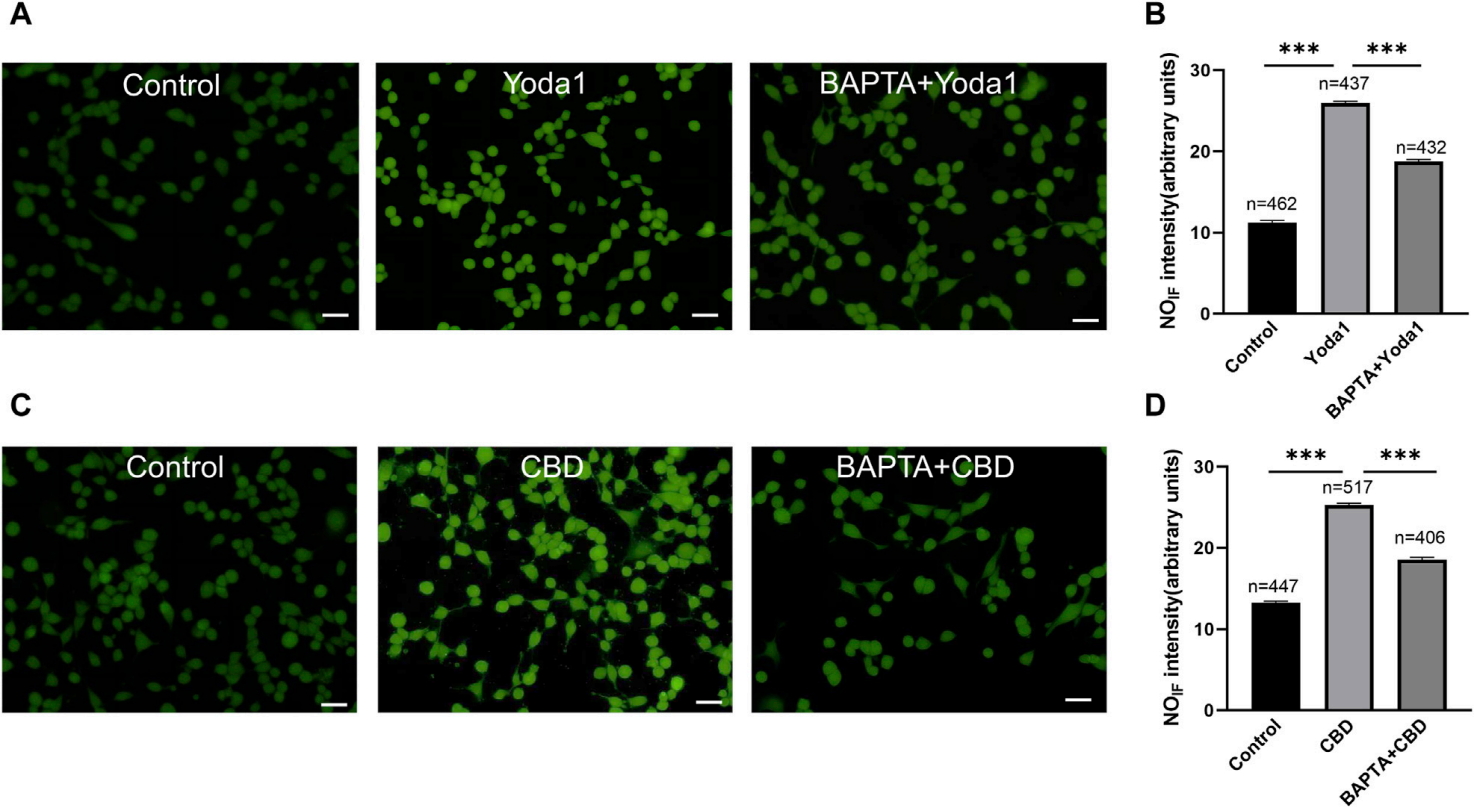
Activation of TRPV2 and Piezo1 induces NO production in cultured urothelial cells. **(A)** Typical images **(A)** showing NO-induced fluorescence (NO_IF_) for cells in the control medium (left), in medium with Yoda1 (30 μM, middle) and in medium with BAPTA (10 μM)+Yoda1 (right). Scale bar = 50 μm. **(B)** Summary data indicate Yoda1 induced change in NO_IF_ and the inhibitory effect of BAPTA. Semi-quantitative analysis of NO_IF_ was performed using imaging analysis software and expressed as fluorescence arbitrary units. **(C,D)** Typical images **(C)** and summary data **(D)** showing CBD (50 μM) induced change in NO_IF_ and the inhibitory effect of BAPTA. The number above each bar correspond to the analyzed cells. ***p < 0.001.

To examine whether activation of TRPV2 and Piezo1 can induce NO production in urothelial cells, a fluorescence NO indicator DAF-2 DA was used to determine NO production and the fluorescence signal intensity was compared in the presence and absence of Piezo1or CBD. To our expectation, application of Yoda-1 (30 μM) and CBD (50 μM) significantly increased the fluorescence signal intensity (Figure 8). Furthermore, pretreatment with BAPTA (10 μM), an cell permeable Ca^2+^ chelator, attenuated the increased effects, suggesting Yoda-1 and CBD induced [Ca^2+^]_i_ increase mediated NO production.

## 4 Discussion

Several previous studies have investigated the expression patterns and functions of MSCs, including TRPV2, TRPV4, Piezo1, and Piezo2, in the urinary bladder. However, to our knowledge, this is the first study to focus on the expression and roles of MSCs in ureteral contractions in particular human ureteral contractions. The main findings are as follows: 1) *Piezo1* and *TRPV2* are the predominantly expressed MSCs at the mRNA level whereas *TRPV4* and *Piezo2* show relatively low expression, 2) agonists of Piezo1 and TRPV2, but not TRPV4, effectively attenuate the frequency of ureteral contractions in a dose-dependent manner, 3) the inhibitory effects of Piezo1 and TRPV2 agonists can be effectively blocked by the selective antagonists, the nitric oxide synthase inhibitor L-NAME, and removal of the mucosa, and 4) Piezo1 and TRPV2 agonists can increase NO production in cultured urothelial cells. Our results suggest that increased production and release of NO from the mucosa induced by the activation of Piezo1 or TRPV2 mediates mechanical stimuli-induced reduction in ureter contractions.

While extensive studies have been conducted on MSCs in urinary bladder (Shabir et al., 2013; Dalghi et al., 2019), the expression patterns of MSCs in human ureter have not been explored in detail (Shabir et al., 2013; Dalghi et al., 2019). All four MSCs (TRPV2, TRPV4, Piezo1, and Piezo2) examined in the present study were expressed in human ureter at both mRNA and protein levels. Ca^2+^ imaging experiments further confirmed the functional expression of TRPV2, TRPV4 and Piezo1 in urothelial cells (Figure 2). However, functional expression of Piezo2 was not investigated due to the lack of specific agonists and antagonists. The current findings are consistent with previous data showing functional expression of TRPV4 in urothelial cells from human ureter (Shabir et al., 2013). To our knowledge, this is the first study to demonstrate the functional expression of Piezo1 and TRPV2 in urothelial cells of human ureter (Dalghi et al., 2019), providing a basis for their modulatory roles in human ureter motility.

Agonists of Piezo1 (Yoda1) and TRPV2 (CBD), but not TRPV4 (GSK), attenuated the frequency of ureteral contractions in a dose-dependent manner and these inhibitory effects were completely blocked by the respective antagonists. These results suggest that activation of Piezo1 and TRPV2, but not TRPV4, plays a critical role in mechano-transduction and response of ureter motility to mechanical stimuli. Notably, inhibitory activity was limited to the frequency of ureteral contractions, with no effects on amplitude of the phasic contractions, suggesting that activation of Piezo1 and TRPV2 specifically affect the pacemaker mechanisms of the ureter.

Unexpectedly, no effects of the TRPV4 agonist were observed, even though GSK evoked a Ca^2+^ increase in urothelial cells, similar to Piezo1 and TRPV2 agonists. The reasons for the lack of impact are unclear. This finding could not be attributed to ineffectiveness of TRPV4 agonist GSK, since GSK affected the Ca^2+^ level in urothelial cells [Figure 2], but is potentially a result of different downstream signals after activation of individual MSCs. For example, TRPV4 activation induces the release of inhibitory transmitters (NO), but these effects could be counteracted by the released excitatory transmitters (ATP, Ach, PGF2). On the other hand, activation of Piezo1 or TRPV2 may evoke lower amounts of excitatory transmitters that are unable to counteract the inhibition. This theory should be tested by measuring the transmitter contents after MSCs activation in urothelial cells in the future.

We propose that activation of Piezo1 and TRPV2 channels on urothelial cells, but not other cell types, mediates the inhibitory effects of agonists based on a number of observations: 1) immunofluorescence experiments demonstrated abundant expression of MSCs in the urothelium (Figure 1B), 2) these MSCs were functional in primary cultured urothelial cells (Figures 2, 3), and most importantly 3) the inhibitory roles of Piezo1 and TRPV2 agonist were lost in denuded ureters (Figure 5). These findings further confirmed the theory that urothelial cells play active modulatory roles in urinary tract function (Canda et al., 2007; Sellers et al., 2018). However, the potential roles of Piezo1 and TRPV2 in other cell types in suburothelial layers, such as interstitial cells (ICs) or sensory nerve terminals, cannot be excluded, since majority of suburothelial ICs and sensory nerves are removed in the denuded ureters. Moreover, data from the current and earlier studies validated the functional expression of Piezo1 and TRPV2 in sub-urothelial ICs (Zhao et al., 2021) and sensory afferents (Lee et al., 2011).

In response to various stimuli, urothelial cells release signaling molecules, such as ATP, NO, PGs and Ach, to enhance or inhibit smooth muscle contractions (Canda et al., 2007; Birder and Andersson, 2013). Consistent with previous reports in ureter (Lee et al., 2011), we observed no effects of Ach [Figure 6A]. Also be consistent with previous study (Morita et al., 1994; Iselin et al., 1997; Lang et al., 2002), ATP played an excitatory role in ureteral contractions [Figure 6B], while application of PGE1, PGE2 and NO donor (SNAP) exerted inhibitory effects [Figures 6C–E]. These findings imply that the inhibitory role of Piezo1 and TRPV2 agonists may be mediated through release of NO or PGs. However, blockage of PG production with a non-selective COX inhibitor did not attenuate inhibition effects of Piezo1 or TRPV2 agonists, suggesting no involvement of the PG pathways. In contrast, pretreatment with L-NAME, a NOS inhibitor, completely blocked the suppressive activity of Piezo1 and TRPV2 agonists, clearly suggesting that NO release contributes to the inhibitory effects. This conclusion was further evidenced by an increased NO production in cultured urothelial cells after treatment with Piezo1 and TRPV2 agonists (Figure 8) and supported by literature report which showed the urothelium is the major site of NO production (Mastrangelo et al., 2003). Our results further support the hypothesis that NO-mediated signal represents a major inhibitory pathway in ureter mobility (Canda et al., 2007).

In physiological or pathological conditions such as obstruction, stent placement, stones, the ureter faces various mechanical stimuli (stretch, pressure, urine flow, and other forces), which induce changes in the frequency of ureteral contractions. For instance, stent insertion initially increased peristaltic activity, which was later reduced or terminated (Venkatesh et al., 2005). In a swine model, normal peristalsis was absent 24 h following ureteral stenting and restored on day 5 (Park and Venkatesh, 2016). In another study, peristaltic activity of porcine ureter increased immediately in response to stent placement, but was decreased after 4–5 h, and was markedly reduced or completely abolished after 1 week (Venkatesh et al., 2005). After removal of obstructions, a brief or long-lasting period of diminished or complete lack of contraction is commonly reported (Vereecken, 1976). To date, few studies have evaluated the mechanisms underlying mechanical stimuli-induced alterations in contractions of the human ureter. Our collective findings indicate that activation of Piezo1 or TRPV2 and successive production and release of NO from the mucosa constitute the important mechanisms involved in reduction of ureteral contractions under the above conditions.

Our study also has limitations. Firstly, we did not record the ureter circumferential force but only the longitudinal force was measured. This may explain the lack of effect of TRPV4 on the contractions. Because these two groups of smooth muscle contract at different times and may evoke different channels. Secondly, we did not examine the roles of mechanical channels in physiological and pathological relevant mechanical stimuli, such as higher pressure or overstretch evoked contractions.

## 5 Conclusion

In summary, we provide evidence that activation of Piezo1 or TRPV2 channels in mucosa play important modulation role in human ureter motility. Our results also provide valuable insights into the mechanisms contributing to mechanical stimuli-induced reduction in ureteral motility under pathological conditions, such as stent or obstruction. Considering that human ureter is the optimal model for ureter pharmacology, Piezo1 and TRPV2 channels may present promising pharmacological targets to improve ureteral stone passage as well as treatment of stent-associated symptoms.

## Data Availability

The raw data supporting the conclusion of this article will be made available by the authors, without undue reservation.

## Supplementary material

SUPPLEMENTARY FIGURE S1 The Knockdown of TRPV2 mRNA attenuates cannabidiol evoked the increase in [Ca^2+^]_i_ in urothelial cells. **(A)** knockdown of TRPV2 was induced by using small interfering RNAs (siRNAs). Gene-specific siRNAs, along with the scrambled siRNA, were designed and synthesized by General Biol (Anhui, China). The *TRPV2* siRNA final sequences were 5′-CUU​CUU​AAA​CUU​CCU​GUG​UAA-3′) (sense) and scrambled siRNA were (5′-ACG​CGU​AAC​GCG​GGA​AUU​U-3′). Treatment with TRPV2 siRNA (Si-TRPV2) reduced the mRNA expression levels of *TRPV2* determined by RT-qPCR analysis to 4.4% of that seen in the untreated cells (Control). Data is the summary from 5 experiments for each group. **(B)** Knockdown TRPV2 expression attenuates cannabidiol evoked the increase in [Ca^2+^]_i_. (a) is the typical recording, and (b) is the summary data. The number above each bar is the cell number recorded. ** *p* < 0.01.

## Abbreviations

Ach, acetylcholine; ATP, Adenosine triphosphate; CBD, Cannabidiol; DMSO, dimethyl sulfoxide; MSCs, Mechano-sensitive channels; NANC, non-adrenergic non-cholinergic; NKA, Neurokinin A; NO, nitric oxide; PBS, phosphate-buffered saline; PGE1, prostaglandin E1; PGE2, prostaglandin E2; PGF2, prostaglandin F2; SNAP, S-Nitroso-N-acetyl-DL-penicillamine; TRPV, transient receptor potential vanilloid.
