# Supplementary figures and images for: Activation of Piezo1 or TRPV2 channels inhibits human ureteral contractions via NO release from the mucosa
Source: Front Pharmacol. 2024 Jun 26;15:1410565. doi: 10.3389/fphar.2024.1410565 (PMC11233528; doi:10.3389/fphar.2024.1410565)

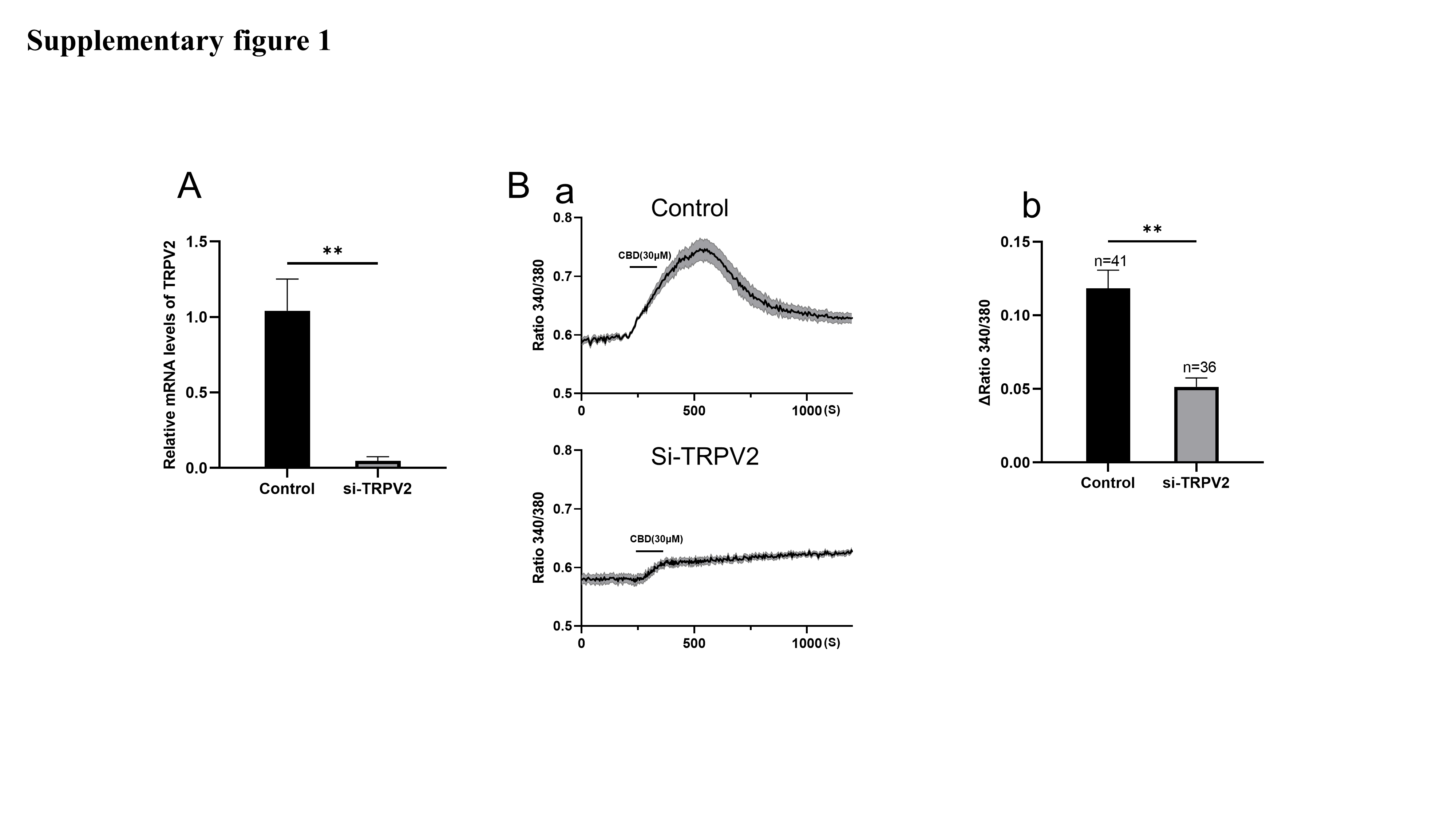

Supplement: Supplementary file 1 [file Image1.tif]
